# 8-{(*E*)-[(4-Chloro­phen­yl)imino]­meth­yl}-1,1,7,7-tetra­methyl-1,2,3,5,6,7-hexa­hydro­pyrido[3,2,1-*ij*]quinolin-9-ol

**DOI:** 10.1107/S1600536813012579

**Published:** 2013-05-15

**Authors:** Esen Nur Kantar, Yavuz Köysal, Nesuhi Akdemir, Ayşen Alaman Ağar, Mustafa Serkan Soylu

**Affiliations:** a Department of Physics, Faculty of Arts and Sciences, Ondokuz Mayıs University, TR-55139 Samsun, Turkey; b Yesilyurt Demir Celik Vocational School, Ondokuz Mayıs University, TR-55139 Samsun, Turkey; c Department of Chemistry, Amasya Faculty of Arts and Sciences, Ondokuz Mayıs University, 05000 Amasya, Turkey; d Department of Chemistry, Faculty of Arts and Sciences, Ondokuz Mayıs University, Kurupelit, 55139 Samsun, Turkey; e Department of Physics, Faculty of Arts and Sciences, Giresun University, Giresun, Turkey

## Abstract

The title Schiff base, C_23_H_27_ClN_2_O adopts the phenol–imine tautomeric form, with an intra­molecular O—H⋯N hydrogen bond, which generates an *S*(6) ring motif. Three C atoms of the heterocyclic moiety of the hexa­hydro­pyrido­quinoline unit, as well as the two methyl groups bonded to one of these C atoms, are disordered over two set of sites, with anoccupancy ratio of 0.740 (4):0.260 (4).

## Related literature

For a related structure, see: Kantar *et al.* (2012 ▶). For hydrogen-bond motifs, see: Bernstein *et al.* (1995 ▶). For ring conformational parameters, see: Cremer & Pople (1975 ▶)
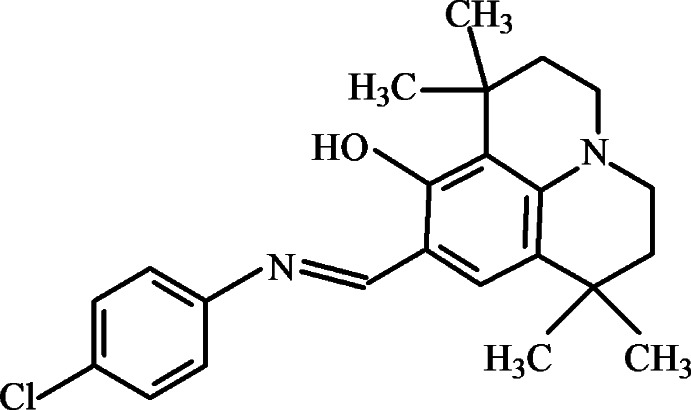


## Experimental

### 

#### Crystal data

C_23_H_27_ClN_2_O
*M* *_r_* = 382.92
Triclinic, 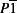

*a* = 6.4716 (3) Å
*b* = 9.8468 (6) Å
*c* = 16.9206 (7) Å
α = 75.438 (4)°
β = 80.307 (4)°
γ = 75.863 (4)°
*V* = 1005.42 (9) Å^3^
*Z* = 2
Mo *K*α radiation
μ = 0.21 mm^−1^
*T* = 293 K
0.30 × 0.10 × 0.10 mm

#### Data collection

Oxford Diffraction SuperNova (Dual, Cu at zero, Eos) diffractometer
Absorption correction: multi-scan (*CrysAlis PRO*; Oxford Diffraction, 2007 ▶) *T*_min_ = 0.947, *T*_max_ = 0.975
5887 measured reflections
3651 independent reflections
2959 reflections with *I* > 2σ(*I*)
*R*_int_ = 0.021

#### Refinement

*R*[*F* ^2^ > 2σ(*F* ^2^)] = 0.055
*wR*(*F* ^2^) = 0.142
*S* = 1.08
3651 reflections
270 parameters
H-atom parameters constrained
Δρ_max_ = 0.51 e Å^−3^
Δρ_min_ = −0.25 e Å^−3^

### 

Data collection: *CrysAlis PRO* (Oxford Diffraction, 2007 ▶); cell refinement: *CrysAlis PRO*; data reduction: *CrysAlis PRO*; program(s) used to solve structure: *SHELXS97* (Sheldrick, 2008 ▶); program(s) used to refine structure: *SHELXL97* (Sheldrick, 2008 ▶); molecular graphics: *ORTEP-3 for Windows* (Farrugia, 2012 ▶); software used to prepare material for publication: *WinGX* (Farrugia, 2012 ▶).

## Supplementary Material

Crystal structure: contains datablock(s) I, global. DOI: 10.1107/S1600536813012579/lr2103sup1.cif

Structure factors: contains datablock(s) I. DOI: 10.1107/S1600536813012579/lr2103Isup2.hkl

Supplementary material file. DOI: 10.1107/S1600536813012579/lr2103Isup3.cml

Additional supplementary materials:  crystallographic information; 3D view; checkCIF report

## Figures and Tables

**Table 1:** Hydrogen-bond geometry (Å, °)

*D*—H⋯*A*	*D*—H	H⋯*A*	*D*⋯*A*	*D*—H⋯*A*
O13—H13⋯N8	0.98	1.65	2.563 (2)	155

**Fig. 1.**
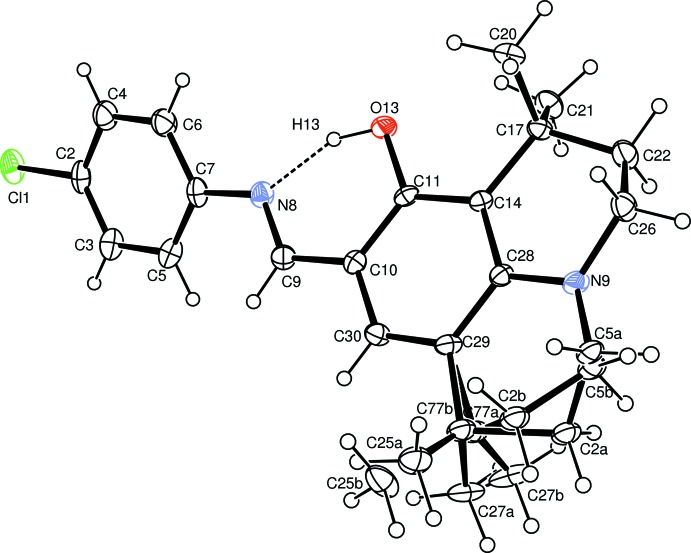
A view of (I), with the displacement ellipsoids drawn at the 30% probability level .

## supplementary crystallographic information

### Comment

As can see in Fig. 1, the molecule of the title compound, C_23_H_27_N_2_OCl, exhibits an intra-molecular O13—H13···N8 [*d*(N···O) 2.563 (3) Å] hydrogen bond which generates an S(6) ring motif (Bernstein, *et al.*, 1995). The compound adopts the phenol-imine tautomeric form. The C7—N8—C9—C10 torsion angle is 177,14 (5)°. The C11—O13 [1.352 (2) Å] bond length is in agreement with our related study of 5-Diethylamino-2-[(*E*)-(2,4-dimethoxyphenyl) iminomethyl ]phenol, (Kantar *et al.*, 2012). The part A (sof=0.740) of the disordered six-membered ring [N9/C28/C29/C31/C32/C33] adopts a half-chair conformation, with puckering parameters: Q = 0.506 (5) Å, θ = 56.1 (5)°, φ = 84.8 (7); while the part B (sof= 0.260) exhibits an envelope conformation with puckering parameters: Q = 0.510 (15) Å, θ = 58.9 (15)°, φ = 236.0 (17)°. Finally the [N9/C26/C22/C17/C14/C28] ring exhibits a half-chair conformation with puckering parameters: Q = 0.490 (3) Å, θ = 124.5 (3)°, φ = 270.9 (3)° (Cremer & Pople, 1975).

### Experimental

The title compound was prepared by refluxing a mixture containing 5-nitro-2-thiophene-carboxaldehyde (0.157 g 1.0 mmol) and 4-Chloroaniline (0.185 g 1.0 mmol) in 20 ml of ethanol for 4 hours. Crystals suitable for X-ray analysis were obtained by slow evaporation from ethanol (yield 63%; m.p 129–131 ^o^C).

### Refinement

The disordered C31 and C33 atoms have to be keeped isotropic during the refinement. All H, except de one bonded to the oxygen atom, were fixed geometrically and refined using a riding model with bond distances 0.93-0.97 Å and U_iso_(H)=1.5U_eq_(C_methyl_) and 1.2 for the remaining hydrogen atoms.

### Crystal data

**Table:**

C_23_H_27_ClN_2_O	*Z* = 2
*M**_r_* = 382.92	*F*(000) = 408
Triclinic, *P*1	*D*_x_ = 1.265 Mg m^−^^3^
Hall symbol: -P 1	Mo *K*α radiation, λ = 0.71073 Å
*a* = 6.4716 (3) Å	Cell parameters from 2478 reflections
*b* = 9.8468 (6) Å	θ = 3.3–28.1°
*c* = 16.9206 (7) Å	µ = 0.21 mm^−^^1^
α = 75.438 (4)°	*T* = 293 K
β = 80.307 (4)°	Block, orange
γ = 75.863 (4)°	0.30 × 0.10 × 0.10 mm
*V* = 1005.42 (9) Å^3^	

### Data collection

**Table:**

Oxford Diffraction SuperNova (Dual, Cu at zero, Eos) diffractometer	3651 independent reflections
Radiation source: SuperNova (Mo) X-ray Source	2959 reflections with *I* > 2σ(*I*)
Mirror monochromator	*R*_int_ = 0.021
Detector resolution: 16.2413 pixels mm^-1^	θ_max_ = 25.5°, θ_min_ = 3.3°
ω scans	*h* = −7→6
Absorption correction: multi-scan (*CrysAlis PRO*; Oxford Diffraction, 2007)	*k* = −11→11
*T*_min_ = 0.947, *T*_max_ = 0.975	*l* = −20→21
5887 measured reflections	

### Refinement

**Table:**

Refinement on *F*^2^	Primary atom site location: structure-invariant direct methods
Least-squares matrix: full	Secondary atom site location: difference Fourier map
*R*[*F*^2^ > 2σ(*F*^2^)] = 0.055	Hydrogen site location: inferred from neighbouring sites
*wR*(*F*^2^) = 0.142	H-atom parameters constrained
*S* = 1.08	*w* = 1/[σ^2^(*F*_o_^2^) + (0.0583*P*)^2^ + 0.4649*P*] where *P* = (*F*_o_^2^ + 2*F*_c_^2^)/3
3651 reflections	(Δ/σ)_max_ < 0.001
270 parameters	Δρ_max_ = 0.51 e Å^−^^3^
0 restraints	Δρ_min_ = −0.25 e Å^−^^3^

### Special details

**Table:**

Geometry. All esds (except the esd in the dihedral angle between two l.s. planes) are estimated using the full covariance matrix. The cell esds are taken into account individually in the estimation of esds in distances, angles and torsion angles; correlations between esds in cell parameters are only used when they are defined by crystal symmetry. An approximate (isotropic) treatment of cell esds is used for estimating esds involving l.s. planes.
Refinement. Refinement of *F*^2^ against ALL reflections. The weighted *R*-factor *wR* and goodness of fit *S* are based on *F*^2^, conventional *R*-factors *R* are based on *F*, with *F* set to zero for negative *F*^2^. The threshold expression of *F*^2^ > σ(*F*^2^) is used only for calculating *R*-factors(gt) *etc*. and is not relevant to the choice of reflections for refinement. *R*-factors based on *F*^2^ are statistically about twice as large as those based on *F*, and *R*- factors based on ALL data will be even larger.

### Fractional atomic coordinates and isotropic or equivalent isotropic displacement parameters (Å^2^)

**Table:**

	*x*	*y*	*z*	*U*_iso_*/*U*_eq_	Occ. (<1)
Cl1	−0.34788 (11)	0.21739 (8)	0.48881 (4)	0.0504 (2)	
O13	0.5458 (3)	0.20809 (16)	0.78056 (10)	0.0319 (4)	
H13	0.4430	0.2230	0.7410	0.085 (11)*	
N8	0.2753 (3)	0.3204 (2)	0.67532 (12)	0.0320 (5)	
N9	0.8858 (3)	0.5185 (2)	0.86809 (12)	0.0304 (5)	
C10	0.4526 (3)	0.4605 (2)	0.72697 (13)	0.0260 (5)	
C14	0.7036 (3)	0.3581 (2)	0.83166 (13)	0.0226 (5)	
C28	0.7385 (3)	0.4979 (2)	0.82400 (13)	0.0246 (5)	
C11	0.5687 (3)	0.3411 (2)	0.78026 (13)	0.0241 (5)	
C29	0.6219 (3)	0.6195 (2)	0.77113 (14)	0.0270 (5)	
C30	0.4837 (3)	0.5960 (2)	0.72526 (14)	0.0286 (5)	
H30	0.4068	0.6749	0.6912	0.034*	
C9	0.3045 (4)	0.4438 (3)	0.67669 (14)	0.0305 (5)	
H9	0.2281	0.5253	0.6443	0.037*	
C26	1.0326 (4)	0.3939 (3)	0.90908 (15)	0.0344 (6)	
H26A	1.1006	0.4211	0.9480	0.041*	
H26B	1.1438	0.3573	0.8689	0.041*	
C3	−0.2026 (4)	0.3834 (3)	0.56288 (15)	0.0396 (6)	
H3	−0.3225	0.4538	0.5475	0.047*	
C2	−0.1648 (4)	0.2534 (3)	0.54194 (14)	0.0375 (6)	
C20	0.9757 (4)	0.1232 (3)	0.85168 (15)	0.0349 (6)	
H20A	0.9121	0.0974	0.8118	0.052*	
H20B	1.0283	0.0386	0.8915	0.052*	
H20C	1.0925	0.1683	0.8247	0.052*	
C17	0.8073 (3)	0.2277 (2)	0.89482 (14)	0.0268 (5)	
C4	0.0146 (4)	0.1472 (3)	0.56321 (16)	0.0436 (7)	
H4	0.0386	0.0593	0.5485	0.052*	
C6	0.1565 (4)	0.1746 (3)	0.60659 (16)	0.0414 (6)	
H6	0.2770	0.1041	0.6213	0.050*	
C5	−0.0594 (4)	0.4092 (3)	0.60749 (15)	0.0369 (6)	
H5	−0.0858	0.4964	0.6232	0.044*	
C7	0.1227 (4)	0.3052 (3)	0.62866 (14)	0.0319 (6)	
C21	0.6364 (4)	0.1482 (3)	0.94807 (15)	0.0369 (6)	
H21A	0.5219	0.2156	0.9703	0.055*	
H21B	0.7004	0.0768	0.9922	0.055*	
H21C	0.5805	0.1026	0.9149	0.055*	
C22	0.9105 (4)	0.2793 (3)	0.95351 (15)	0.0370 (6)	
H22A	0.7997	0.3169	0.9934	0.044*	
H22B	1.0076	0.1984	0.9831	0.044*	
C31A	0.6373 (6)	0.7726 (4)	0.7709 (3)	0.0261 (12)*	0.740 (4)
C32A	0.7660 (5)	0.7750 (3)	0.8375 (2)	0.0349 (9)	0.740 (4)
H32A	0.6760	0.7673	0.8898	0.042*	0.740 (4)
H32B	0.8115	0.8656	0.8250	0.042*	0.740 (4)
C33A	0.9657 (7)	0.6498 (5)	0.8444 (3)	0.0270 (13)*	0.740 (4)
H33A	1.0553	0.6542	0.7922	0.032*	0.740 (4)
H33B	1.0499	0.6548	0.8855	0.032*	0.740 (4)
C25A	0.7433 (7)	0.8359 (4)	0.6858 (2)	0.0409 (10)	0.740 (4)
H25A	0.8866	0.7811	0.6772	0.061*	0.740 (4)
H25B	0.7480	0.9337	0.6828	0.061*	0.740 (4)
H25C	0.6616	0.8324	0.6443	0.061*	0.740 (4)
C27A	0.4126 (6)	0.8685 (4)	0.7847 (3)	0.0413 (10)	0.740 (4)
H27A	0.3304	0.8730	0.7415	0.062*	0.740 (4)
H27B	0.4269	0.9634	0.7844	0.062*	0.740 (4)
H27C	0.3409	0.8286	0.8367	0.062*	0.740 (4)
C31B	0.6651 (17)	0.7763 (12)	0.7479 (8)	0.023 (3)*	0.260 (4)
C32B	0.8988 (16)	0.7638 (10)	0.7656 (6)	0.038 (3)	0.260 (4)
H32C	0.9239	0.8578	0.7636	0.045*	0.260 (4)
H32D	1.0017	0.7213	0.7251	0.045*	0.260 (4)
C33B	0.922 (2)	0.6647 (14)	0.8544 (8)	0.031 (4)*	0.260 (4)
H33C	1.0648	0.6575	0.8675	0.037*	0.260 (4)
H33D	0.8213	0.7128	0.8933	0.037*	0.260 (4)
C25B	0.6297 (15)	0.8709 (9)	0.6668 (6)	0.048 (3)	0.260 (4)
H25D	0.7062	0.8223	0.6246	0.072*	0.260 (4)
H25E	0.6806	0.9568	0.6619	0.072*	0.260 (4)
H25F	0.4792	0.8955	0.6610	0.072*	0.260 (4)
C27B	0.5165 (15)	0.8384 (9)	0.8190 (6)	0.064 (4)	0.260 (4)
H27D	0.3701	0.8610	0.8077	0.097*	0.260 (4)
H27E	0.5563	0.9239	0.8232	0.097*	0.260 (4)
H27F	0.5320	0.7686	0.8699	0.097*	0.260 (4)

### Atomic displacement parameters (Å^2^)

**Table:**

	*U*^11^	*U*^22^	*U*^33^	*U*^12^	*U*^13^	*U*^23^
Cl1	0.0556 (5)	0.0669 (5)	0.0414 (4)	−0.0331 (4)	−0.0197 (3)	−0.0059 (3)
O13	0.0394 (9)	0.0221 (8)	0.0378 (10)	−0.0104 (7)	−0.0103 (8)	−0.0053 (7)
N8	0.0338 (11)	0.0336 (11)	0.0317 (11)	−0.0126 (9)	−0.0055 (9)	−0.0068 (9)
N9	0.0298 (10)	0.0235 (10)	0.0434 (12)	−0.0069 (8)	−0.0137 (9)	−0.0098 (9)
C10	0.0246 (11)	0.0288 (12)	0.0260 (12)	−0.0064 (9)	−0.0020 (9)	−0.0085 (10)
C14	0.0212 (10)	0.0206 (11)	0.0262 (11)	−0.0034 (9)	−0.0012 (9)	−0.0072 (9)
C28	0.0221 (11)	0.0236 (12)	0.0298 (12)	−0.0032 (9)	−0.0044 (9)	−0.0095 (10)
C11	0.0262 (11)	0.0201 (11)	0.0283 (12)	−0.0093 (9)	0.0028 (9)	−0.0088 (9)
C29	0.0245 (11)	0.0207 (11)	0.0364 (13)	−0.0038 (9)	−0.0045 (10)	−0.0074 (10)
C30	0.0263 (12)	0.0246 (12)	0.0332 (13)	−0.0032 (10)	−0.0062 (10)	−0.0038 (10)
C9	0.0308 (12)	0.0308 (13)	0.0305 (13)	−0.0097 (10)	−0.0023 (10)	−0.0055 (10)
C26	0.0323 (13)	0.0372 (14)	0.0360 (14)	−0.0103 (11)	−0.0118 (11)	−0.0041 (11)
C3	0.0283 (12)	0.0593 (18)	0.0351 (14)	−0.0126 (12)	−0.0016 (11)	−0.0156 (13)
C2	0.0386 (14)	0.0589 (17)	0.0226 (12)	−0.0272 (13)	−0.0047 (10)	−0.0055 (12)
C20	0.0351 (13)	0.0233 (12)	0.0403 (14)	−0.0003 (10)	−0.0045 (11)	−0.0017 (11)
C17	0.0277 (12)	0.0221 (12)	0.0289 (12)	−0.0059 (9)	−0.0037 (10)	−0.0017 (9)
C4	0.0555 (17)	0.0433 (16)	0.0400 (15)	−0.0201 (13)	−0.0168 (13)	−0.0067 (12)
C6	0.0477 (15)	0.0380 (15)	0.0431 (15)	−0.0134 (12)	−0.0173 (13)	−0.0051 (12)
C5	0.0330 (13)	0.0505 (16)	0.0333 (13)	−0.0155 (12)	0.0016 (11)	−0.0175 (12)
C7	0.0313 (13)	0.0444 (15)	0.0229 (12)	−0.0178 (11)	−0.0034 (10)	−0.0028 (11)
C21	0.0380 (14)	0.0364 (14)	0.0333 (14)	−0.0136 (11)	−0.0034 (11)	0.0030 (11)
C22	0.0369 (14)	0.0380 (14)	0.0359 (14)	−0.0087 (11)	−0.0119 (11)	−0.0020 (11)
C32A	0.044 (2)	0.0231 (17)	0.042 (2)	−0.0125 (15)	−0.0010 (16)	−0.0130 (15)
C25A	0.050 (3)	0.029 (2)	0.044 (2)	−0.0176 (19)	0.000 (2)	−0.0031 (18)
C27A	0.036 (2)	0.0156 (17)	0.072 (3)	−0.0011 (16)	−0.0018 (19)	−0.0152 (18)
C32B	0.049 (6)	0.023 (5)	0.045 (6)	−0.018 (4)	−0.016 (5)	0.002 (4)
C25B	0.054 (8)	0.027 (6)	0.067 (8)	−0.024 (6)	−0.030 (7)	0.016 (5)
C27B	0.056 (9)	0.030 (7)	0.107 (13)	−0.013 (6)	0.020 (8)	−0.032 (8)

### Geometric parameters (Å, º)

**Table:**

Cl1—C2	1.750 (2)	C6—H6	0.9300
O13—C11	1.352 (2)	C5—C7	1.391 (3)
O13—H13	0.9827	C5—H5	0.9300
N8—C9	1.280 (3)	C21—H21A	0.9600
N8—C7	1.420 (3)	C21—H21B	0.9600
N9—C28	1.384 (3)	C21—H21C	0.9600
N9—C33A	1.448 (5)	C22—H22A	0.9700
N9—C26	1.453 (3)	C22—H22B	0.9700
N9—C33B	1.470 (13)	C31A—C32A	1.517 (5)
C10—C30	1.390 (3)	C31A—C25A	1.535 (6)
C10—C11	1.422 (3)	C31A—C27A	1.545 (5)
C10—C9	1.446 (3)	C32A—C33A	1.550 (5)
C14—C11	1.396 (3)	C32A—H32A	0.9700
C14—C28	1.419 (3)	C32A—H32B	0.9700
C14—C17	1.540 (3)	C33A—H33A	0.9700
C28—C29	1.432 (3)	C33A—H33B	0.9700
C29—C30	1.372 (3)	C25A—H25A	0.9600
C29—C31A	1.534 (4)	C25A—H25B	0.9600
C29—C31B	1.576 (11)	C25A—H25C	0.9600
C30—H30	0.9300	C27A—H27A	0.9600
C9—H9	0.9300	C27A—H27B	0.9600
C26—C22	1.508 (3)	C27A—H27C	0.9600
C26—H26A	0.9700	C31B—C25B	1.470 (15)
C26—H26B	0.9700	C31B—C32B	1.561 (14)
C3—C2	1.368 (4)	C31B—C27B	1.561 (15)
C3—C5	1.393 (3)	C32B—C33B	1.581 (16)
C3—H3	0.9300	C32B—H32C	0.9700
C2—C4	1.389 (4)	C32B—H32D	0.9700
C20—C17	1.530 (3)	C33B—H33C	0.9700
C20—H20A	0.9600	C33B—H33D	0.9700
C20—H20B	0.9600	C25B—H25D	0.9600
C20—H20C	0.9600	C25B—H25E	0.9600
C17—C22	1.527 (3)	C25B—H25F	0.9600
C17—C21	1.543 (3)	C27B—H27D	0.9600
C4—C6	1.379 (3)	C27B—H27E	0.9600
C4—H4	0.9300	C27B—H27F	0.9600
C6—C7	1.385 (3)		
			
C11—O13—H13	105.1	H21A—C21—H21B	109.5
C9—N8—C7	121.8 (2)	C17—C21—H21C	109.5
C28—N9—C33A	120.1 (2)	H21A—C21—H21C	109.5
C28—N9—C26	118.91 (18)	H21B—C21—H21C	109.5
C33A—N9—C26	114.7 (2)	C26—C22—C17	112.2 (2)
C28—N9—C33B	117.4 (5)	C26—C22—H22A	109.2
C33A—N9—C33B	12.9 (6)	C17—C22—H22A	109.2
C26—N9—C33B	121.9 (5)	C26—C22—H22B	109.2
C30—C10—C11	117.72 (19)	C17—C22—H22B	109.2
C30—C10—C9	120.4 (2)	H22A—C22—H22B	107.9
C11—C10—C9	121.9 (2)	C32A—C31A—C29	111.9 (3)
C11—C14—C28	117.93 (19)	C32A—C31A—C25A	110.3 (3)
C11—C14—C17	119.97 (19)	C29—C31A—C25A	107.6 (3)
C28—C14—C17	122.10 (18)	C32A—C31A—C27A	107.8 (3)
N9—C28—C14	120.20 (19)	C29—C31A—C27A	111.2 (3)
N9—C28—C29	119.01 (19)	C25A—C31A—C27A	108.0 (3)
C14—C28—C29	120.80 (19)	C31A—C32A—C33A	111.2 (3)
O13—C11—C14	119.77 (19)	C31A—C32A—H32A	109.4
O13—C11—C10	118.44 (18)	C33A—C32A—H32A	109.4
C14—C11—C10	121.79 (19)	C31A—C32A—H32B	109.4
C30—C29—C28	118.1 (2)	C33A—C32A—H32B	109.4
C30—C29—C31A	120.8 (2)	H32A—C32A—H32B	108.0
C28—C29—C31A	121.0 (2)	N9—C33A—C32A	106.4 (3)
C30—C29—C31B	114.9 (4)	N9—C33A—H33A	110.4
C28—C29—C31B	126.1 (4)	C32A—C33A—H33A	110.4
C31A—C29—C31B	14.6 (4)	N9—C33A—H33B	110.4
C29—C30—C10	123.4 (2)	C32A—C33A—H33B	110.4
C29—C30—H30	118.3	H33A—C33A—H33B	108.6
C10—C30—H30	118.3	C31A—C25A—H25A	109.5
N8—C9—C10	122.1 (2)	C31A—C25A—H25B	109.5
N8—C9—H9	118.9	H25A—C25A—H25B	109.5
C10—C9—H9	118.9	C31A—C25A—H25C	109.5
N9—C26—C22	109.41 (19)	H25A—C25A—H25C	109.5
N9—C26—H26A	109.8	H25B—C25A—H25C	109.5
C22—C26—H26A	109.8	C31A—C27A—H27A	109.5
N9—C26—H26B	109.8	C31A—C27A—H27B	109.5
C22—C26—H26B	109.8	H27A—C27A—H27B	109.5
H26A—C26—H26B	108.2	C31A—C27A—H27C	109.5
C2—C3—C5	119.2 (2)	H27A—C27A—H27C	109.5
C2—C3—H3	120.4	H27B—C27A—H27C	109.5
C5—C3—H3	120.4	C25B—C31B—C32B	109.6 (9)
C3—C2—C4	121.5 (2)	C25B—C31B—C27B	112.2 (9)
C3—C2—Cl1	119.7 (2)	C32B—C31B—C27B	105.4 (8)
C4—C2—Cl1	118.8 (2)	C25B—C31B—C29	121.7 (8)
C17—C20—H20A	109.5	C32B—C31B—C29	107.0 (7)
C17—C20—H20B	109.5	C27B—C31B—C29	99.6 (8)
H20A—C20—H20B	109.5	C31B—C32B—C33B	106.4 (9)
C17—C20—H20C	109.5	C31B—C32B—H32C	110.4
H20A—C20—H20C	109.5	C33B—C32B—H32C	110.4
H20B—C20—H20C	109.5	C31B—C32B—H32D	110.4
C22—C17—C20	110.06 (19)	C33B—C32B—H32D	110.4
C22—C17—C14	109.37 (18)	H32C—C32B—H32D	108.6
C20—C17—C14	110.74 (18)	N9—C33B—C32B	117.3 (9)
C22—C17—C21	106.53 (19)	N9—C33B—H33C	108.0
C20—C17—C21	109.19 (19)	C32B—C33B—H33C	108.0
C14—C17—C21	110.87 (18)	N9—C33B—H33D	108.0
C6—C4—C2	118.7 (3)	C32B—C33B—H33D	108.0
C6—C4—H4	120.6	H33C—C33B—H33D	107.2
C2—C4—H4	120.6	C31B—C25B—H25D	109.5
C4—C6—C7	121.1 (2)	C31B—C25B—H25E	109.5
C4—C6—H6	119.4	H25D—C25B—H25E	109.5
C7—C6—H6	119.4	C31B—C25B—H25F	109.5
C7—C5—C3	120.4 (2)	H25D—C25B—H25F	109.5
C7—C5—H5	119.8	H25E—C25B—H25F	109.5
C3—C5—H5	119.8	C31B—C27B—H27D	109.5
C6—C7—C5	119.1 (2)	C31B—C27B—H27E	109.5
C6—C7—N8	116.4 (2)	H27D—C27B—H27E	109.5
C5—C7—N8	124.4 (2)	C31B—C27B—H27F	109.5
C17—C21—H21A	109.5	H27D—C27B—H27F	109.5
C17—C21—H21B	109.5	H27E—C27B—H27F	109.5
			
C33A—N9—C28—C14	162.8 (3)	C2—C4—C6—C7	−0.2 (4)
C26—N9—C28—C14	12.0 (3)	C2—C3—C5—C7	1.6 (4)
C33B—N9—C28—C14	177.2 (7)	C4—C6—C7—C5	0.9 (4)
C33A—N9—C28—C29	−17.5 (4)	C4—C6—C7—N8	178.2 (2)
C26—N9—C28—C29	−168.3 (2)	C3—C5—C7—C6	−1.7 (4)
C33B—N9—C28—C29	−3.1 (7)	C3—C5—C7—N8	−178.7 (2)
C11—C14—C28—N9	−174.10 (19)	C9—N8—C7—C6	156.8 (2)
C17—C14—C28—N9	6.6 (3)	C9—N8—C7—C5	−26.1 (4)
C11—C14—C28—C29	6.2 (3)	N9—C26—C22—C17	61.8 (3)
C17—C14—C28—C29	−173.14 (19)	C20—C17—C22—C26	78.4 (3)
C28—C14—C11—O13	174.30 (19)	C14—C17—C22—C26	−43.4 (3)
C17—C14—C11—O13	−6.4 (3)	C21—C17—C22—C26	−163.3 (2)
C28—C14—C11—C10	−5.8 (3)	C30—C29—C31A—C32A	169.0 (3)
C17—C14—C11—C10	173.53 (19)	C28—C29—C31A—C32A	−6.0 (4)
C30—C10—C11—O13	−177.8 (2)	C31B—C29—C31A—C32A	−120.9 (19)
C9—C10—C11—O13	3.1 (3)	C30—C29—C31A—C25A	−69.7 (4)
C30—C10—C11—C14	2.3 (3)	C28—C29—C31A—C25A	115.3 (3)
C9—C10—C11—C14	−176.8 (2)	C31B—C29—C31A—C25A	0.4 (17)
N9—C28—C29—C30	177.2 (2)	C30—C29—C31A—C27A	48.4 (4)
C14—C28—C29—C30	−3.1 (3)	C28—C29—C31A—C27A	−126.6 (3)
N9—C28—C29—C31A	−7.7 (4)	C31B—C29—C31A—C27A	118.5 (19)
C14—C28—C29—C31A	172.1 (3)	C29—C31A—C32A—C33A	40.8 (4)
N9—C28—C29—C31B	8.8 (7)	C25A—C31A—C32A—C33A	−78.8 (4)
C14—C28—C29—C31B	−171.5 (6)	C27A—C31A—C32A—C33A	163.4 (3)
C28—C29—C30—C10	−0.6 (3)	C28—N9—C33A—C32A	51.5 (4)
C31A—C29—C30—C10	−175.8 (3)	C26—N9—C33A—C32A	−156.5 (3)
C31B—C29—C30—C10	169.1 (5)	C33B—N9—C33A—C32A	−30 (3)
C11—C10—C30—C29	1.0 (3)	C31A—C32A—C33A—N9	−62.5 (4)
C9—C10—C30—C29	−179.8 (2)	C30—C29—C31B—C25B	−21.9 (11)
C7—N8—C9—C10	177.1 (2)	C28—C29—C31B—C25B	146.8 (7)
C30—C10—C9—N8	178.4 (2)	C31A—C29—C31B—C25B	−139 (2)
C11—C10—C9—N8	−2.4 (3)	C30—C29—C31B—C32B	−148.8 (6)
C28—N9—C26—C22	−45.4 (3)	C28—C29—C31B—C32B	20.0 (11)
C33A—N9—C26—C22	162.3 (3)	C31A—C29—C31B—C32B	94 (2)
C33B—N9—C26—C22	150.1 (7)	C30—C29—C31B—C27B	101.7 (6)
C5—C3—C2—C4	−0.9 (4)	C28—C29—C31B—C27B	−89.5 (7)
C5—C3—C2—Cl1	177.92 (18)	C31A—C29—C31B—C27B	−15.3 (14)
C11—C14—C17—C22	−169.2 (2)	C25B—C31B—C32B—C33B	178.3 (9)
C28—C14—C17—C22	10.1 (3)	C27B—C31B—C32B—C33B	57.4 (11)
C11—C14—C17—C20	69.3 (3)	C29—C31B—C32B—C33B	−48.0 (11)
C28—C14—C17—C20	−111.4 (2)	C28—N9—C33B—C32B	−32.1 (13)
C11—C14—C17—C21	−52.1 (3)	C33A—N9—C33B—C32B	73 (3)
C28—C14—C17—C21	127.2 (2)	C26—N9—C33B—C32B	132.6 (8)
C3—C2—C4—C6	0.1 (4)	C31B—C32B—C33B—N9	59.4 (13)
Cl1—C2—C4—C6	−178.7 (2)		

### Hydrogen-bond geometry (Å, º)

**Table:**

*D*—H···*A*	*D*—H	H···*A*	*D*···*A*	*D*—H···*A*
O13—H13···N8	0.98	1.65	2.563 (2)	155
